# Market penetration of Xpert MTB/RIF in high tuberculosis burden countries: A trend analysis from 2014 - 2016

**DOI:** 10.12688/gatesopenres.12842.2

**Published:** 2018-09-18

**Authors:** Danielle Cazabon, Tripti Pande, Sandra Kik, Wayne Van Gemert, Hojoon Sohn, Claudia Denkinger, Zhi Zhen Qin, Brenda Waning, Madhukar Pai

**Affiliations:** 1 McGill International TB Centre, McGill University, Montreal, QC, Canada; 2 Foundation for Innovative New Diagnostics, FIND, Geneva, Switzerland; 3 Stop TB Partnership, Geneva, Switzerland; 4 Bloomberg School of Public Health, Johns Hopkins University, Baltimore, MD, USA; 5 Epidemiology & Biostatistics, McGill University, Montreal, QC, Canada

**Keywords:** tuberculosis, diagnostics, market penetration, Xpert MTB/RIF, access

## Abstract

**Background: **Xpert® MTB/RIF, a rapid tuberculosis (TB) molecular test, was endorsed by the World Health Organization in 2010. Since then, 34.4 million cartridges have been procured under concessional pricing. Although the roll out of this diagnostic is promising, previous studies showed low market penetration.

**Methods: **To assess 3-year trends of market penetration of Xpert MTB/RIF in the public sector, smear and Xpert MTB/RIF volumes for the year 2016 were evaluated and policies from 2014-2016 within 22 high-burden countries (HBCs) were studied. A structured questionnaire was sent to representatives of 22 HBCs. The questionnaires assessed the total smear and Xpert MTB/RIF volumes, number of modules and days of operation of GeneXpert machines in National TB Programs (NTPs). Data regarding the use of NTP GeneXpert machines for other diseases and GeneXpert procurement by other disease control programs were collected. Market penetration was estimated by the ratio of total sputum smear volume for initial diagnosis divided by the number of Xpert MTB/RIF tests procured in the public sector.

**Results: **The survey response rate was 21/22 (95%). Smear/Xpert ratios decreased in 17/21 countries and increased in four countries, since 2014. The median ratio decreased from 32.6 (IQR: 44.6) in 2014 to 6.0 (IQR: 15.4) in 2016. In 2016, the median GeneXpert utilization was 20%, however seven countries (7/19; 37%) were running tests for other diseases on their NTP-procured GeneXpert systems in 2017, such as HIV, hepatitis-C virus (HCV), *Chlamydia trachomatis*, and *Neisseria gonorrhoeae*. Five (5/15; 33%) countries reported GeneXpert procurement by HIV or HCV programs in 2016 and/or 2017.

**Conclusions: **Our results show a positive trend for Xpert MTB/RIF market penetration in 21 HBC public sectors. However, GeneXpert machines were under-utilized for TB, and inadequately exploited as a multi disease technology.

## Introduction

Tuberculosis (TB), caused 1.7 million deaths in 2016, has surpassing human immunodeficiency infection and acquired immune deficiency syndrome (HIV/AIDS) as the leading cause of death. The World Health Organization (WHO) reported 10.4 million new TB cases in 2016, with 87% of them occurring in 30 high TB burden countries (HBCs).

The WHO endorsed the Xpert® MTB/RIF assay (Xpert MTB/RIF; Cepheid, Sunnyvale, CA, USA) for TB in 2010. By the end of 2017, 34.4 million Xpert MTB/RIF reagent cartridges had been procured in the public sector under concessional pricing ^1^. Xpert Ultra, a test with a higher sensitivity than Xpert MTB/RIF, was released in 2017 and also endorsed by the WHO ^2^.

While the roll out of these diagnostic tools is promising, previous analyses from Qin *et al.* in 2014 and Cazabon *et al.* in 2015 showed low market penetration in comparison to the conventional sputum smear microscopy test - there were 32.6 and 9.1 sputum smears for every Xpert MTB/RIF test procured in 2014 and 2015, respectively ^3^,^4^. To assess 3-year trends in the public sector, we evaluated the smear and Xpert MTB/RIF volumes for the year 2016 and studied changes in Xpert MTB/RIF market penetration and policies from 2014–2016 within 22 HBCs.

## Methods

A structured questionnaire ( Supplementary File 1) was created based on two previous studies ^3^,^4^, and sent via e-mail to staff members of Ministries of Health, National TB Programs (NTP), National Reference Laboratories and TB research institutes of 22 HBCs, as defined by WHO prior to 2015 ^5^. A maximum of two staff members were contacted per HBC. A response was taken as consent to participate in this study. Ethics approval was not obtained, as this was a market research study on test volumes, and no human subject data were collected.

The survey included questions about smear volumes (stratified by smears used for initial diagnosis vs. treatment monitoring), the number of Xpert MTB/RIF tests conducted in the country, the number of days of operation of NTP GeneXpert machines, and the number of NTP GeneXpert modules in operation, all for the year 2016. The questionnaire also included questions on implementation of Xpert Ultra, the use of NTP GeneXperts for other diseases (e.g. HIV), and other programme procurement of GeneXpert in the year 2017.

Data on Xpert MTB/RIF cartridge procurement was obtained from Cepheid, via Foundation for Innovative New Diagnostics (FIND) and WHO-recommended TB rapid diagnostics (WRD) algorithms were obtained from WHO ^1^. WRDs include Xpert MTB/RIF and TB-LAMP (loop-mediated isothermal amplification) ^1^; however based on expert knowledge of limited LAMP adoption to date and to allow comparisons with previous years, we assumed WRDs to be represented by Xpert MTB/RIF only.

Data were collected between November 2017 and March 2018. Participants were contacted via e-mail a maximum of three times to respond. If any clarification was required, the participant was contacted.

Xpert MTB/RIF market penetration, estimated by the ratio of total sputum smear volume for initial diagnosis divided by the number of Xpert MTB/RIF tests procured in the public sector, was calculated for 2016 in each country (smear/Xpert ratio; ^4^) and compared to the same ratio in 2014 and 2015. A decreasing ratio signified a greater use of Xpert compared to smear microscopy for the diagnosis of TB. The median smear/Xpert ratio was calculated for 2016 and compared to prior years.

To calculate the percent utilization of GeneXpert for TB diagnosis in the public sector, the actual number of Xpert MTB/RIF tests conducted in 2016 was compared to the full capacity of GeneXpert for TB diagnosis during the same year. Full capacity of GeneXpert for TB diagnosis was calculated using the following assumptions; an 8-hour workday and 2 hours to run each Xpert MTB/RIF test, i.e. 4 tests per module per working day. This was multiplied by the number of operating days of GeneXpert in 2016. Data were entered and tabulated using Excel 2016.

## Results

Data from Cepheid and WHO reports were available for all 22 HBCs. Our survey response rate was 21/22 (95%). All missing values were marked as “not available” (NA) and were removed from descriptive analyses.

**Figure 1.**
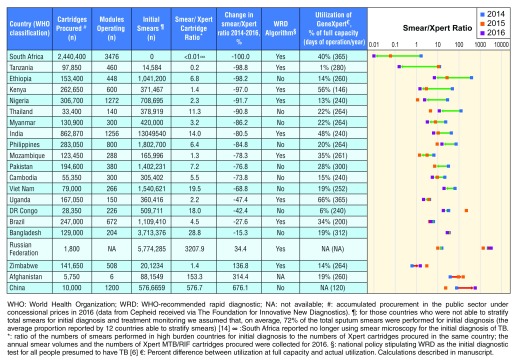
Xpert MTB/RIF market penetration in the public sector of 21 high TB burden countries (2014–2016). South Africa decreased its smear/Xpert ratio by 100%, therefore has achieved the highest market penetration of Xpert MTB/RIF in the public sector. Countries are listed in order of greatest percent decrease in smear/Xpert ratio from 2014–2016. Arrows represent percent changes in smear/Xpert ratios 2014–2016. Trends moving closer to 0 (ratio decreasing) signify a greater use of Xpert compared to smear microscopy for the diagnosis of TB. Trends moving away from 0 (ratio increasing) signify a greater use of smear microscopy compared to Xpert for the diagnosis of TB.

In 2016, 6.9 million Xpert MTB/RIF cartridges had been procured globally in the public sector under concessional pricing, compared to 4.8 million in 2014. In the 22 HBCs that we studied, a total of 5.8 million Xpert MTB/RIF cartridges were procured in 2016. According to the WHO Global TB Report, 10/22 (48%) countries included Xpert MTB/RIF in their national policy stipulating the use of Xpert MTB/RIF as the initial diagnostic test for all people presumed to have TB in 2016, compared to 4/22 (18%) countries in 2015 ^1^,^6^. Overall, smear/Xpert ratios decreased in 17/21 (81%) HBCs and increased in four (19%) from 2014 to 2016. The median ratio decreased from 32.6 (IQR: 44.6) in 2014 to 6.0 (IQR: 15.4) in 2016 ( Figure 1).

Figure 1 shows thirteen (66%) countries had a smear/Xpert ratio <10, five (19%) countries between 10–50, no countries between 51–100 and three (14%) countries had a ratio >100, in 2016. Whereas in 2014, five (23%) countries had a ratio <10, 11 (50%) countries between 10–50, three (14%) countries between 51–100 and three (14%) countries had a ratio of >100. South Africa had the largest decrease in smear/Xpert ratio from 2014–2016.

**Figure 2.**
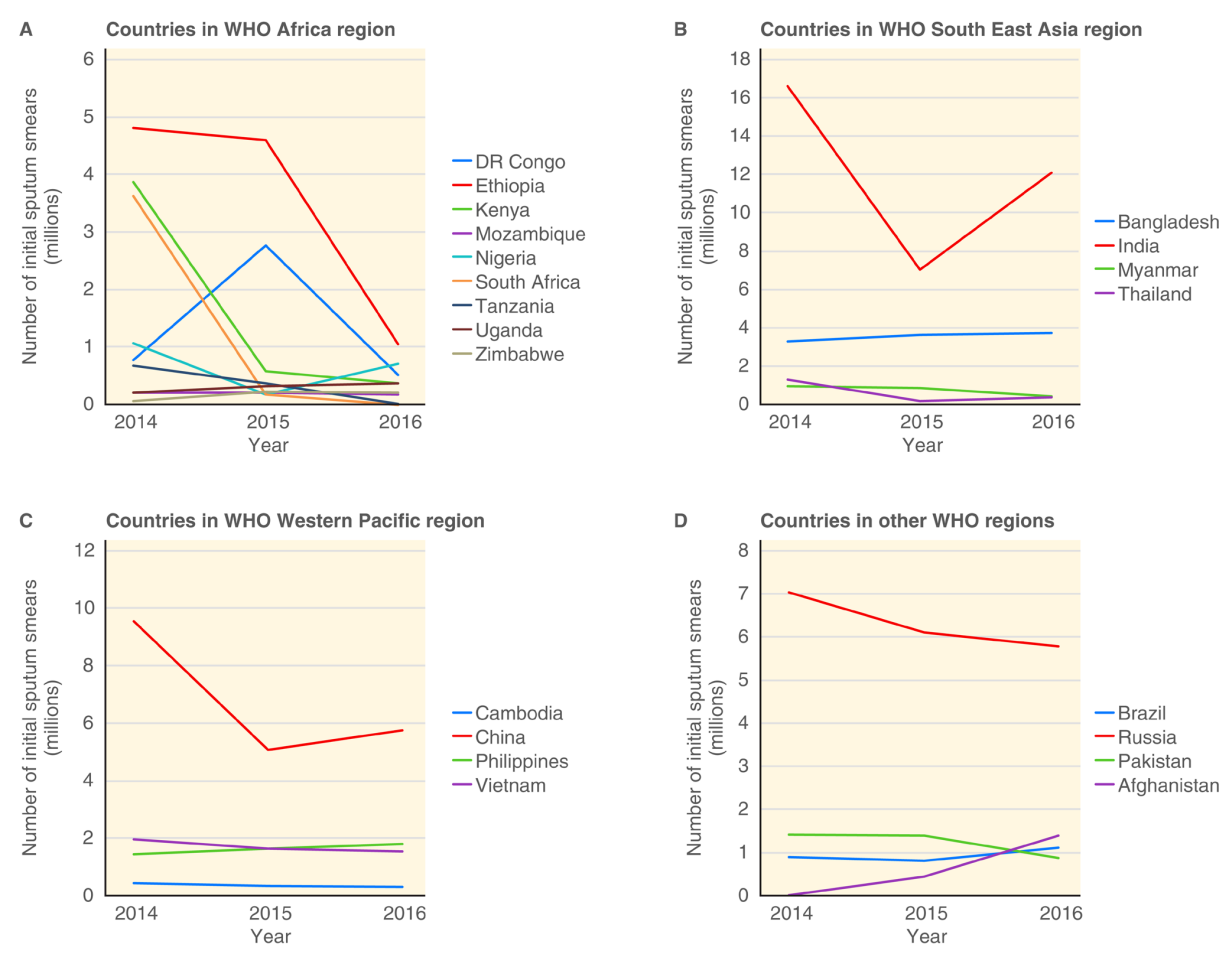
Initial sputum smear microscopy use in World Health Organization (WHO) regions from 2014–2016.

**Figure 3.**
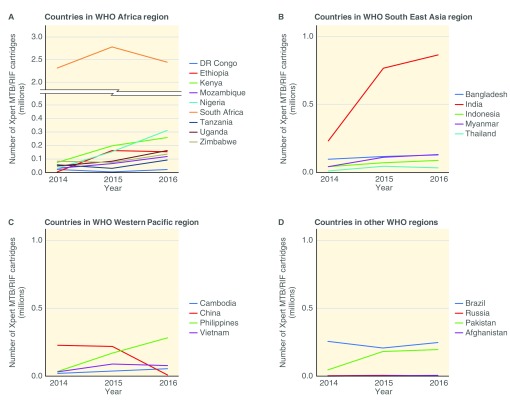
Xpert MTB/RIF cartridge procurement in World Health Organization (WHO) regions from 2014–2016.

Countries in the African region had a median decline of 34% in the number of initial smears conducted between 2014 and 2016. In South East Asia, Western pacific and the other WHO regions, the median changes in initial smears were -41%, -26% and +2%, respectively. Figure 2 and Figure 3 show the trends of initial smears conducted and Xpert MTB/RIF procurement in each country over the period of 2014–2016, respectively. In 2016, the median GeneXpert utilization was 20%.

Table 1 describes other uses of GeneXpert machines in each HBC. Five (5/21; 24%) countries had procured Xpert Ultra in 2017; three (14%) were utilizing it in the public sector and two (10%) had not yet implemented it. Six (29%) countries have no current plans to procure it in 2018 whereas ten (48%) plan to procure it in 2018. In addition to Xpert MTB/RIF, seven countries (7/19; 37%) were also utilizing tests for other diseases on their NTP-procured GeneXperts in 2017, such as HIV, hepatitis-C virus (HCV), *Chlamydia trachomatis*, and *Neisseria gonorrhoeae*. South Africa was utilizing the previously mentioned tests, as well as *Clostridium difficile*; carbapenem resistance; and influenza. GeneXperts have also been procured by other programmes; 5/15 (33%) countries reported procurement by HIV or HCV programmes in 2016 and/or 2017.

**Table 1.** Other public sector utilization of GeneXpert in 21 high tuberculosis (TB) burden countries (2016–2017).

Country (WHO classification)	Use of NTP GeneXpert for diagnosing other diseases (2017)	Other programs procuring GeneXpert machines (2016, 2017)	Implementation of Xpert Ultra cartridges in the public sector (2017)
**South Africa**	HCV VL, HIV-1 VL, HPV, NG/CT, C Diff, Carba R, Influenza	HIV	Procured and in use in the public sector
**India**	No	NA	In discussion *
**Tanzania**	HCV-VL, HIV-1 VL, HIV-1 qual/EID	No	Procured and in use in the public sector, plan to procure in 2018 (private sector)
**Ethiopia**	Piloting (HIV-1 qual/EID and HIV-1 VL)	Yes	Plan to procure in 2018
**Kenya**	No	No	Plan to procure in 2018
**Nigeria**	HCV-VL, HIV-1 VL, HPV, NG/CT	HIV	Plan to procure in 2018
**Thailand**	No	No	Do not have definitive plans
**Myanmar**	No	HIV, HCV	Procured but not yet in use in the public sector ^#^
**Philippines**	No	No	Do not have definitive plans
**Mozambique**	No	No	Plan to procure in 2018
**Pakistan**	HCV VL, HIV-1 VL	No	Procured and in use in the public sector
**Cambodia**	NA	NA	Plan to procure in 2018
**Viet Nam**	No	NA	Plan to procure in 2018
**Uganda**	HIV-VL	NA	Plan to procure in 2018
**DR Congo**	No	No	Do not have definitive plans
**Brazil**	No	No	Plan to procure in 2018
**Bangladesh**	No	No	Do not have definitive plans
**Russian Federation**	NA	NA	Do not have definitive plans
**Zimbabwe**	HIV-1 VL, HIV-1 qual/EID	HIV	Procured, but not yet in use in the public sector
**Afghanistan**	No	No	Plan to procure in 2018
**China**	NA	NA	Do not have definitive plans

WHO: World Health Organization; NTP: National tuberculosis programme; HCV-VL: Hepatitis-C virus- viral load; HIV-VL: human immunodeficiency virus- viral load; HPV: human papilloma virus; NG/CT: *Neisseria gonorrhoeae* (NG) and *Chlamydia trachomatis* (CT); C Diff: *Clostridium difficile*; Carba R: Carbapenem resistance; Influenza: Detection of Flu A and Flu B with 2009 H1N1 Call Out; HIV-1 qual/EID: human immunodeficiency virus qualitative test/Early infant diagnosis; NA: not available; *India is currently discussing whether Xpert Ultra should be implemented within the public sector; # Myanmar is currently using Xpert Ultra in a prevalence survey.

## Discussion

Overall, our data shows that high-burden countries have demonstrated a positive trend towards greater use of Xpert MTB/RIF. South Africa had the largest percent decrease in smear/Xpert ratio from 2014–2016, making it the leader in GeneXpert market penetration. This is underscored by the fact that South Africa did not report any smears for initial diagnosis of TB in 2016.

Despite our 3-year trend analysis having shown an increase in the number of countries that included Xpert MTB/RIF in their national policy as the initial diagnostic test for all people presumed to have TB since 2014, only 48% of countries had it included in 2016. More countries are encouraged to adopt this policy to achieve an increase of TB and drug resistant TB (DR-TB) case detection ^1^. Xpert MTB/RIF may require high upfront and running costs, however it has been shown in India (a high burden MDR-TB country) that the mean total costs (per MDR case initiated on treatment) are lower when using Xpert MTB/RIF for all presumptive TB patients, compared to solely using it for those at risk of DR-TB ^7^. This is particularly relevant for high MDR-TB countries, who represent 17 (77%) of the 22 HBCs.

While most countries showed a positive trend towards greater use of Xpert MTB/RIF, a few showed decreasing Xpert use. A potential explanation for countries with decreasing market penetration trends, (i.e. increasing smear/Xpert ratios), could be the end of Global Fund grants causing a transition to domestic-financing and procurement by NTPs ^8^. For example, China’s Xpert MTB/RIF procurement decreased considerably in 2016 and the number of initial smears increased, suggesting that other diagnostics tests were being used instead of Xpert MTB/RIF. The Global Fund’s substantial co-financing requirements will shift financing and procurement responsibilities towards national programs over the next three years. Close monitoring of the impact on TB diagnostics procurement, utilization, and market penetration will be important to identify and address barriers to access.

Most of the countries in the African region had a decline in the number of initial smears conducted (median= -34%) between 2014 and 2016 ( Figure 2), suggesting that Xpert MTB/RIF is replacing smears for the initial diagnosis of TB in most instances. In other regions, there is a slight increase in the use of initial smears (median= +2%). A possible explanation could be the use of GeneXpert in addition to smear for initial diagnosis, or only using it to test specific populations (e.g. those at risk for multi-drug resistant TB (MDR-TB)). Certain countries, such as India and the Democratic Republic of Congo, showed an overall decline in initial smears between 2014 and 2016, however there were large peaks in 2015. This could be due to variations in reporting from year to year.

All countries in our study were underutilizing GeneXpert for TB testing. This is consistent with results from a study conducted in 18 countries where 63% of sites surveyed had access to Xpert MTB/RIF, but only 4% of TB/HIV co-infected patients had been tested for TB using Xpert MTB/RIF. Further, over 50% of the patients that did not receive a TB test at all were treated at facilities where a GeneXpert was available on-site ^9^.

Full utilization of GeneXpert requires various components such as; familiarization of the technology among health care workers, efficient supply chain, rapid swapping of failed modules, an efficient and wide-reaching sample referral system, and moving away from reliance on empiric treatment, which remain barriers to implementation in certain countries ^9^,^10^.

While certain countries in our study were integrating testing services between disease control programmes, our results elucidate the need for more integration amongst vertical disease programs to increase efficient utilization of GeneXpert, which is a multi-disease platform. For example, NTP-procured GeneXpert machines can be utilized to run other tests, such as Xpert HIV-1 viral load (VL) ^11^. Zimbabwe has recently shown that integration of the GeneXpert platform for MTB/RIF, HIV-1 VL and HIV-1 qualitative test/Early infant diagnosis (qual/EID) was feasible and improved access to these tests in priority populations ^12^. Further, the use of GeneXpert as a multi-disease platform may potentially eliminate redundant purchases of machines thus decreasing equipment costs to health ministries.

There are several limitations to our study. First, data from the private sector and data from commercial procurement of Xpert MTB/RIF by public sector entities in certain countries (due to failure to meet concessional pricing conditions) were not included, thus the smear volumes and Xpert procurement are not representative of country totals. However, data shows that Xpert MTB/RIF prices are high in the private sector, and volumes are likely to be low ^13^. Due to the Initiative for Promoting affordable and Quality TB Tests (IPAQT) in India, Xpert MTB/RIF is available at lower prices in the private sector ^14^. Introducing such initiative in other countries may increase assessibility to Xpert MTB/RIF. Second, cartridge procurement data may not reflect the actual utilisation of Xpert MTB/RIF. Third, to measure the full capacity of utilization for GeneXpert, we used pre-identified assumptions, such as an 8-hour working day. This assumption did not account for the variability in operating hours at different TB laboratories in each country, which could have led to inaccurate estimates. Moreover, we did not account for GeneXperts that may have been used for other diseases. Fourth, it was assumed that two smears were conducted for initial diagnosis of TB in all countries. While this may not be accurate for all countries, this had a limited effect on the trends of market penetration of MTB/RIF in each country over time. Fifth, we did not collect data on financing and procurement efficiency to estimate how these may have impacted utilization. Next, it is possible that survey data was reported differently from one year to the next which could have led to inaccurate estimates of initial smears and thus, smear/Xpert ratios. Next, in 2015 the WHO expanded its high TB burden list to include an additional ten countries ^15^. Our review was unable to analyze results from these countries as our response rate was too low (1/10; 10%). Finally, data availability was limited in some countries (e.g. Tanzania).

## Conclusion

Our results show a positive trend for Xpert MTB/RIF market penetration in 21 HBC public sectors, however there remains underutilization of GeneXpert machines, and insufficient use of GeneXpert as a multi-disease platform. There is great scope for countries to improve this and optimize the usage and impact of novel, multi-disease technologies like GeneXpert. HBCs should go beyond specific high-risk groups to a broader use of the technology as a frontline TB test, and use the platform to reach universal drug-susceptibility targets. HBCs should also go beyond using GeneXpert for just TB, and exploit the platform to deliver a variety of tests included in the WHO Essential Diagnostics List.

## Data availability

All data presented in this manuscript is available on Open Science Framework (OSF) under the repository “Smear Xpert analysis 2018”: https://doi.org/10.17605/osf.io/vsxe8 ^16^.

S1 Raw data contains all responses posed by the questionnaire for 21 HBCs. It also contains Xpert MTB/RIF procurement data which was provided by FIND.

Data are available under the terms of the Creative Commons Zero "No rights reserved" data waiver (CC0 1.0 Public domain dedication).

## Notes

No data available from WHO Global TB Report on the use of WRD as an initial diagnostic for all individuals with presumed TB in country algorithms for the year 2014.

## Supplementary material

Supplementary File 1: Structured questionnaire.
