# Supplementary material for: Market penetration of Xpert MTB/RIF in high tuberculosis burden countries: A trend analysis from 2014 - 2016
Source: Gates Open Res. 2018 Sep 18;2:35. Originally published 2018 Jul 25. [Version 2] doi: 10.12688/gatesopenres.12842.2 (PMC6139378; doi:10.12688/gatesopenres.12842.2)
Supplement: Supplementary file 1 [file gatesopenres-2-13954-s0000.tgz › 923056e8-4494-4959-90ff-742b4e6fc7ef.pdf]

Survey of national policies and practices for Xpert® MTB/RIF implementation

This is a collaborative study by McGill International Tuberculosis Centre, Stop TB Partnership and FIND. We had previously conducted two studies evaluating how Xpert® MTB/RIF, a World Health Organization-endorsed molecular test, is being implemented in 22 high tuberculosis (TB) burden countries by NTPs (National TB Programmes) and their partners/Non-Governmental Organizations(NGOs). They were published in the European Respiratory Journal and are available at the following: Qin et al., 2015 and Cazabon et al., 2017. Currently, we are conducting a third survey to analyze the trends of Xpert® MTB/RIF implementation over time and would be most grateful if you could kindly fill in the questions below for your country. We anticipate that this questionnaire will take approximately 10-15 minutes.

Examples of results are filled out in the tables below, to be used as guidance. The results of this survey will be summarized and presented by country. They will ultimately be published in a peer-reviewed journal. Participants names will remain anonymous, and all information will remain confidential.

Please return your answers within the coming 2 weeks to danielle.cazabon@mail.mcgill.ca. We greatly appreciate your support for this project.

**required*

Respondent Information

1. **Country ***

   _____

2. **Respondent ***
   Your identity is required only for administrative purposes.

   - ☐ NTP manager or staff
   - ☐ NGO (partner with NTP)
   - ☐ Country Coordinating Mechanism of the Global Fund
   - ☐ Other (please specify) _____

Sputum-smear microscopy usage in your country

Please fill in the far-right column with your country information with data from Jan 1, 2016 to Dec 31, 2016.

	EXAMPLE	YOUR COUNTRY
**3. In 2016, approximately how many microscopy labs were there in your country in the NTP or public sector?** *	240	____
**4. In 2016, approximately how many smears were done in the public sector (including all presumed TB, and including both initial and repeat smears done for treatment monitoring)?** *	4,565,856	____
**5. Can you break down the above number by smears done for initial diagnosis and smears done for treatment monitoring?** * **(In the absence of exact numbers, you may provide percentages)**	☐ YES<br>☒ NO<br><br>**If yes, please provide the numbers**:<br><br>**Initial:** data n/a<br><br>**Treatment monitoring:** data n/a<br><br>**%Initial/ %Monitoring:**<br><br>40%:60%	☐ YES<br>☐ NO<br><br>**If yes, please provide the numbers**:<br><br>**Initial:**<br>____<br><br>**Treatment monitoring:**<br>____<br><br>**%Initial/ %Monitoring:**<br>____
**6. In 2016, how many individuals (people, not tests) with presumed TB underwent smear testing in your country in the public sector?** *	900 000	____
**7. How many smears per patient does the NTP recommend to do for initial diagnosis? [i.e. what is the NTP policy on number of smears required per patient?]** *	2 smears in smear testing algorithm	____

Xpert® MTB/RIF roll-out in your country

8. **What is your current national policy or algorithm for Xpert® MTB/RIF testing in your country? * ***(Choose all that apply)*

Xpert is recommended as the initial diagnostic test for:

- ☐ All adults presumed to have TB
- ☐ People at risk of HIV-associated TB
- ☐ People at risk of drug-resistant TB
- ☐ Children presumed to have TB
- ☐ Extrapulmonary TB (EPTB)
  If possible, please specify the specimen type: _____
- ☐ Other (please specify) _____

9. **As per your country policy, in which tier of the healthcare system is Xpert® MTB/RIF currently available in the public sector? ***
(Choose all that apply)

- ☐ Peripheral (or community) laboratory (e.g. microscopy centers)[1]
- ☐ Intermediate (or regional) laboratory [2]
- ☐ National / reference lab(s) [3]
- ☐ Other (please specify) _____

10. **In 2016, how many days of the year were GeneXpert laboratories operating in the public sector (on average)**? * _____

11. **How many Xpert® MTB/RIF tests were performed in the public sector in 2016**? *
- ☐ Total number of MTB/RIF test performed: _____
- ☐ Information not available

12. **How many GeneXpert machines were operating in the public sector and used by TB programmes in 2016? ***
Please fill out the table below. If no GeneXpert machines were operating in the public sector and used by TB programmes in 2016, please write 0.

Type of GeneXpert Machine	Total operating in TB programmes in 2016
GeneXpert I	_____
GeneXpert II	_____
Gene Xpert IV	_____
Gene Xpert XVI	_____

---

[1] Prepares, stains, and examines smears, may use Xpert MTB/RIF, TB-LAMP or LF-LAM according to national diagnostic algorithms.

[2] Performs all of the functions of a Level 1 laboratory plus LPA and culture.

[3] Performs all of the functions of Level 1 and Level 2 laboratories plus performs identification of MTB, first- and second-line DST.

13. **Is your country implementing *Xpert Ultra* cartridges in the public sector?** *

☐ Procured, but not yet in use in the public sector
☐ Procured and in use in the public sector
☐ Plan to procure in 2018 (please specify date, if known): _____
☐ Do not have definitive plans to procure in 2018
☐ Other: _____

14. **Are tests other than Xpert MTB/RIF or Xpert Ultra MTB/RIF run on the GeneXpert machines that were procured for the NTP?** *
(Choose all that apply)

☐ *Xpert HCV Viral Load*
☐ *Xpert HIV-1 Viral Load*
☐ *Xpert HIV-1 Qual/ EID (early infant diagnosis)*
☐ *Xpert HPV*
☐ *Xpert NG/CT (Chlamydia trachomatis (CT) and Neisseria gonorrhoeae (NG))*
☐ *Xpert Ebola*
☐ Other, please specify: _____
☐ No other tests are run on NTP procured GeneXpert machines

Additional Diagnostics roll-out in your country

15. **In the table below, please indicate whether other public sector programs (other than NTP) procured GeneXpert machines in 2016 and 2017.***
Please specify the number of machines procured for each disease, for each year. If no machines were procured by other public sector programmes, please write 0.

Disease	Year 2016	Year 2017
☐ HIV:	_____	_____
☐ HCV:	_____	_____
☐ Other (please specify):	_____	_____

16. **Questions 16 a-c refer to TB-LAMP (Loop Mediated Isothermal Amplification)** *
   a. ***Does your country's national policy include TB-LAMP tests for diagnosing TB?***

☐ Yes

☐ No

☐ Information not available

b. ***Is your country currently utilizing TB-LAMP for diagnosing TB in the public sector?*** *

☐ Yes
☐ No
☐ Information not available

c. ***Is your country planning to procure TB-LAMPS under the new round of Global Fund Concept Notes?*** *

☐ Yes
☐ No
☐ Information not available

17. **Question 17 a - c refer to <u>TB –LAM Ag Test (LF-LAM)</u>***

a. ***Does your country's national policy include LF – LAM for diagnosing TB?***

☐ Yes
☐ No
☐ Information not available

b. ***Is your country currently utilizing LF – LAM for diagnosing TB in the public sector?***

☐ Yes
☐ No
☐ Information not available

c. ***Is your country planning to procure LF – LAM under the new round of Global Fund Concept Notes?***

☐ Yes
☐ No
☐ Information not available

Comments

1. **Please provide any comments, questions, and clarifications in the space provided below.**

_____

On behalf of McGill International TB Centre, Stop TB Partnership and FIND we would like to sincerely thank you for your contribution to this important project!

If there any questions, please email us at danielle.cazabon@mail.mcgill.ca or tripti.pande@mail.mcgill.ca.
