# Supplementary material for: Market penetration of Xpert MTB/RIF in high tuberculosis burden countries: A trend analysis from 2014 - 2016
Source: Gates Open Res. 2018 Sep 18;2:35. Originally published 2018 Jul 25. [Version 2] doi: 10.12688/gatesopenres.12842.2 (PMC6139378; doi:10.12688/gatesopenres.12842.2)
Supplement: Peer review history

# Peer review: Referee response for version 1

**DOI:** 10.21956/gatesopenres.13923.r26585

**Published:** 2018-08-30

**Authors:** Melissa L. Mugambi, Mickey Urdea

**Affiliations:** 1 Department of Global Health, University of Washington, Seattle, WA, USA; 2 Halteres Associates, Emeryville, CA, USA

This is a well written article which is highly relevant to the donor audience and suggests that more work is needed to stimulate demand beyond concessional pricing efforts. It also begs the question – why does it take years before we see the type of scale we want to see with diagnostic devices? What type of business or partnership models can help address the issue of scale? In light of the need to stimulate country demand, it might be helpful for the authors to consider making a bold statement about impact e.g. we estimate that if utilization increased by x% we would expect a y% increase in TB case detection. Stepping beyond a performance assessment and actually stating what closing the gap (increasing market penetration) will do to TB case detection is an important statement that must be made. Similarly, the resource perspective will be helpful as well – what will it cost to close the gap? 

 The authors raise a key limitation to the study: “it is possible that survey data was reported differently from one year to the next which could have led to inaccurate estimates of initial smears and thus, smear/Xpert ratios.” It is important to note that the quality of the data is probably a big issue and while it cannot feasibly be addressed in this paper, it would be interesting and helpful to explore the validity of this data in more country-specific analyses.

We have read this submission. We believe that we have an appropriate level of expertise to confirm that it is of an acceptable scientific standard.

# Response

**Published:** 2018-09-14

**Authors:** Madhukar Pai

**Affiliations:** McGill University, Canada

Thank you for your comments. 

 1. While we appreciate this comment, we do not feel we can accurately state what amount of increased market penetration will close the gap in TB case detection. Nor what will it cost to close the gap. The data collected does not allow us to estimate this, since the goal of this study is to assess trends over time. 

 2. We agree!

# Peer review: Referee response for version 1

**DOI:** 10.21956/gatesopenres.13923.r26586

**Published:** 2018-08-20

**Authors:** Cesar Ugarte Gil

**Affiliations:** 1 School of Medicine Alberto Hurtado, Institute of Tropical Medicine Alexander von Humboldt, Cayetano Heredia University (UPCH), Lima, Peru

Interesting manuscript, I have a few comments:

1. Heterogeneity among study sites:  Some of the sites (i.e. South Africa) had more experience in Xpert's implementation, and maybe some other sites not. Also, maybe some sites didn't complete the process of implementation.  This info can be added in Table 1.
2. Utilization of full capacity:  Besides Uganda, all countries are below 50%.  What is the reasons/hypothesis behind that?.  Also, as a previous reviewer said, the assumption of 8 working hours can fit in a perfect system, in many of these centers, laboratory technicians are multi-tasking or are not open all day (or didn't have electricity all day). Taking this account can be helpful to see if is a real under-utilization of Xpert.
3. Use of other tests (LAM and LAMP):  In the structured questionnaire there are some questions about these tests, is possible to add some info about these answers? The use of these tests can change the screening process.  Maybe is not implemented yet, but this can change the Xpert utilization and (maybe) is possible to forecast Xpert use in these settings.

I have read this submission. I believe that I have an appropriate level of expertise to confirm that it is of an acceptable scientific standard.

# Response

**Published:** 2018-09-14

**Authors:** Madhukar Pai

**Affiliations:** McGill University, Canada

Than you for your comments. 

1. Implementation progress of Xpert is represented by the smear/Xpert ratio, which is already included in Table 1. Factors that could have affected implementation in certain countries are included in the text. 

2. Addressed this already, based off of Reviewer 1's comments. Included as a limitation. 

3. Great point. We decided after designing the questionnaire, however, that this study would focus only on GeneXpert and not include results on TB LAM or LAMP.

# Peer review: Referee response for version 1

**DOI:** 10.21956/gatesopenres.13923.r26587

**Published:** 2018-08-08

**Authors:** Erica Lessem

**Affiliations:** 1 Treatment Action Group (TAG), New York City, NY, USA

This is a very important article and provides useful information about trends in utilization of WHO-endorsed test GeneXpert MTB/RIF at the country level, which can help guide donor policy as well as advocacy for this test and other interventions. 

 A couple minor questions/suggestions:

- Are authors confident that the smears being reported are only for the initial test (as asked)? I am not sure how countries record those data to know if this would be very reliable, so that could be a potential limitation. 
- It is noted that private sector prices are lower in India; I think it would be helpful for less informed readers to briefly explain why (I am assuming this is because of IPAQT)
- Could the authors also suggest what the ideal smear to Xpert ratio for initial test should be in their view? Is it really no smears at all, or are there caveats? This could help contextualize the ranges given in Fig 1. 
- Also, if I am reading Fig 3 right, Xpert procurement decreased in South Africa from 2015 to 2016 even though the smear to Xpert ratio has also decreased. Do authors have an explanation for this interesting finding, e.g. does this mean that South Africa has reached saturation and is bending curve downwards, or have they stopped reaching as many people in need of dx? 
- I understand given preceding analysis the focus on HBCs as defined by WHO prior to 2015, but I think it could be very interesting to look at the additional HBCs on the 2016-2020 list, even if you won't be able to have the previous data points for historical comparison, it can at least provide a snapshot of where the countries are now and be a baseline for further analyses moving forward. 

Great article and very important overall.

I have read this submission. I believe that I have an appropriate level of expertise to confirm that it is of an acceptable scientific standard.

# Response

**Published:** 2018-09-14

**Authors:** Madhukar Pai

**Affiliations:** McGill University, Canada

Reviewer 2:

Thank you for your comments.

1. Great point, however we feel as if this is covered by the following limitations that were included in the text:

- It is possible that survey data was reported differently from one year to the next which could have led to inaccurate estimates of initial smears and thus, smear/Xpert ratios.
- Data availability was limited in some countries, which could have led to imprecise estimates.

2. This has been added to the text and reference added.

3. This is indicated in the caption of the figure: “Trends moving closer to 0 (ratio decreasing) signify a greater use of Xpert compared to smear microscopy for the diagnosis of TB. Trends moving away from 0 (ratio increasing) signify a greater use of smear microscopy compared to Xpert for the diagnosis of TB".

4. Great point brought up by the reviewer. Authors are unsure of the reasons for decrease, but this could be explored further in the next study.

5. We tried to collect data from additional countries but the response rate was very low (1/10 responded after contacting them a maximum of 3 times), therefore we decided to exclude this data. This information has been added to the text.

# Peer review: Referee response for version 1

**DOI:** 10.21956/gatesopenres.13923.r26588

**Published:** 2018-08-08

**Authors:** Zibusiso Ndlovu

**Affiliations:** 1 Southern Africa Medical Unit, Medecins Sans Frontieres (MSF), Cape Town, South Africa

**Introduction**

1. Paragraph 2: First mention of Xpert must be in full (GeneXpert) and the rest could be Xpert
2. Paragraph 2: Put the name ‘ *reagent cartridges*’ after Xpert MTB/Rif
3. Paragraph 3: the last sentence of needs paraphrasing, it has the repetitive use of the word ‘ *assess*’. You can use synonyms like: evaluate etc 

**Methods**

1. 3rd paragraph: revise the first sentence and replace ‘by’ with ‘from’=> ‘Data on Xpert MTB/Rif cartridge procurement was obtained…. **from**’
2. 3rd paragraph: revise the first sentence and add ‘TB’. ‘….and WHO recommended diagnostic algorithms (WRD) for **TB **were obtained from WHO’..
3. Last paragraph: I tend to disagree with the assumption of ‘full capacity’ utilization Xpert is 16tests per Xpert per day. This is not pragmatic and it is setting the trend high because lab techs multi-task and also, its only feasible ‘on average’ to conduct 4 tests in the morning, another 4 tests after tea break and another 4 tests after lunch hours….so total possible pragmatically is 12 tests per day.. Your assumption of 16 tests per day, then provides a bias toward a high under-utilization of devices yet the counterfactual is not possible.  

**Results**

1. They do not flow very well from theme-to-theme; you need to work on these. 
2. 2nd paragraph: put % next to 5.8million (to show the proportion ordered in HBC vs total ordered)
3. 2nd paragraph: remove the word ‘as shown’; and combine the two succeeding sentences. Also, put IQR [x-y] and not mention the words Q1 and Q3 as they make it clumsy; also, remove the % for the countries (17/21 (%)). Also add the median ration of the increases among the 4 countries. 
4. Paragraph 4: why do you now have a denominator of 19/19 (100%) yet your survey was to 22 countries? Why not mention the median ‘capacity utilization’ instead of telling us that 100% were not using to full capacity? These now seem like your own inferences
5. Paragraph 4: (last sentence)=>The statement describing the countries who had recommended and using Xpert as the first diagnostic test, must appear within the first part of your results where you give us a background as to the study countries and who had implemented what etc… currently, your results are not organized.
6. Name of Figure must not be paragraph long. 
7. Last paragraph: 
  a.    Avoid using the word ‘running’, its not scientific

**Discussion**

1. Discussion must also flow very well in sync with results chronology. After the small introduction, proceed by discussing why some countries still haven’t endorsed Xpert as the first test and how many they are compared to 2014 and rationale. 
2. Paragraph 3: revise the 1st sentence and add; ‘.. ***number***’ of initial smears conducted…. Also, do we have a % of the average decline in the number of SM done vs Xpert?

I have read this submission. I believe that I have an appropriate level of expertise to confirm that it is of an acceptable scientific standard.

# Response

**Published:** 2018-09-14

**Authors:** Madhukar Pai

**Affiliations:** McGill University, Canada

Introduction: All comments have been addressed in the revised text.  

 Methods:

 The 2 first points have been addressed in the revised text. 

 Point 3. This point is well taken. However, this is an assumption and has been acknowledged as a limitation. 

 Results: 

 The flow has been revised based on the reviewer's suggestions.

 The grammar/sentence changes have been addressed based on reviewer suggestions. 

 The denominator has become 19 because 2 countries did not respond to this question. These were excluded, and this is explained in the text. 

 Changed the % under utilizing to median % under utilization. 

 Name of the figure is short. The description of the figure remains in the caption.

 Discussion: 

 The flow has been revised based on reviewer's suggestions. 

 Have included the median change in number of initial smears for each region in the results.
